# Uncovering the Resistance Mechanism of *Mycobacterium tuberculosis* to Rifampicin Due to RNA Polymerase H451D/Y/R Mutations From Computational Perspective

**DOI:** 10.3389/fchem.2019.00819

**Published:** 2019-12-03

**Authors:** Qianqian Zhang, Xiaoli An, Hongli Liu, Shuo Wang, Tong Xiao, Huanxiang Liu

**Affiliations:** ^1^School of Pharmacy, Lanzhou University, Lanzhou, China; ^2^State Key Laboratory of Applied Organic Chemistry and Department of Chemistry, Lanzhou University, Lanzhou, China

**Keywords:** tuberculosis, rifampicin, drug resistance, molecular dynamics simulation, molecular mechanics generalized-Born surface area, dynamic network analysis

## Abstract

Tuberculosis is still one of the top 10 causes of deaths worldwide, especially with the emergence of multidrug-resistant tuberculosis. Rifampicin, as the most effective first-line antituberculosis drug, also develops resistance due to the mutation on *Mycobacterium tuberculosis* (Mtb) RNA polymerase. Among these mutations, three mutations at position 451 (H451D, H451Y, H451R) are associated with high-level resistance to rifampicin. However, the resistance mechanism of Mtb to rifampicin is still unclear. In this work, to explore the resistance mechanism of Mtb to rifampicin due to H451D/Y/R mutations, we combined the molecular dynamics simulation, molecular mechanics generalized-Born surface area calculation, dynamic network analysis, and residue interactions network analysis to compare the interaction change of rifampicin with wild-type RNA polymerase and three mutants. The results of molecular mechanics generalized-Born surface area calculations indicate that the binding free energy of rifampicin with three mutants decreases. In addition, the dynamic network analysis and residue interaction network analysis show that when H451 was mutated, the interactions of residue 451 with its adjacent residues such as Q438, F439, M440, D441, and S447 disappeared or weakened, increasing the flexibility of binding pocket. At the same time, the disappearance of hydrogen bonds between R613 and rifampicin caused by the flipping of R613 is another important reason for the reduction of binding ability of rifampicin in H451D/Y mutants. In H451R mutant, the mutation causes the binding pocket change too much so that the position of rifampicin has a large movement in the binding pocket. In this study, the resistance mechanism of rifampicin at the atomic level is proposed. The proposed drug-resistance mechanism will provide the valuable guidance for the design of antituberculosis drugs.

## Introduction

Tuberculosis (TB), an infectious disease caused by *Mycobacterium tuberculosis* (Mtb), is the leading cause from a single infectious agent worldwide. Mtb, a pathogenic bacterium species of the family *Mycobacteriaceae*, can attack the lung of people and spread in the population through the droplets from the throat of an active TB infection patient (Mishra and Surolia, 2018). Millions of people continue to fall sick with TB each year. The 2018 WHO Global Tuberculosis Report (Organization, 2018) estimated 10.0 million new cases of TB and 1.6 million deaths in 2017.

Although it spreads widely, TB is preventable and curable. The 6-month short-course regimen with a combination of four anti-TB drugs (rifampicin, isoniazid, pyrazinamide, and ethambutol) has been used as a standard treatment for active, drug-susceptible TB patients over the past decades (Service and Council, 1981; Chang et al., 2018; Seid et al., 2018; Tiberi et al., 2018). Furthermore, the risk of relapse is generally below 5% reported among drug-susceptible TB patients after treated with standard 6-month regimens in clinical trials (Chang et al., 2006). However, the multidrug-resistant tuberculosis (MDR-TB), which at least resists to both rifampicin and isoniazid, had been emerged in the early 1990s due to multiple factors (He et al., 2008; Sandgren et al., 2009; Ahmad et al., 2018).

Rifampicin, one of the most effective anti-TB drugs, has been used as the first-line treatment in drug-susceptible TB patients, and it is also effective against initial isoniazid resistance (Mitchison and Nunn, 1986). Unfortunately, rifampicin resistance in Mtb arises due to the residues' mutations on its molecular target, *Mycobacterium tuberculosis* RNA polymerase (Mtb-RNAP). More than 95% of the rifampin-resistant strains have mutations in a small region defined “rifampicin resistance-determining region” in Mtb-RNAP (Morlock et al., 2000; Zaw et al., 2018). The most common mutation in rifampicin resistance-determining region are S456, H451, and D441, corresponding to S531, H526, and D516 in *Escherichia coli*, respectively. Studies have shown that 70% of rifampicin-resistant clinical isolates have point mutation in two residues (S456 and H451) (Morlock et al., 2000), and H451 is most usually substituted for Asp (D), Tyr (Y), and Arg (R) (Telenti et al., 1993; Caws et al., 2006; Ma et al., 2006; Chikaonda et al., 2017; Wu and Hilliker, 2017). As early as 1995, the *in vitro* activity experiment of rifampicin by Bodmer et al. (1995) had been demonstrated that H451D/Y/R mutations could cause high-level resistance to rifampicin. After more than 20 years, the level and frequency of resistance to rifampicin are also increasing.

In 2017, there is about 558,000 new cases of rifampicin-resistant tuberculosis (RR-TB), of which 82% are MDR-TB and about 230,000 deaths from MDR/RR-TB (Organization, 2018). Currently, although MDR/RR-TB can be cured with the second-line drugs (e.g., fluoroquinolone and an injectable aminoglycoside), poor efficiency, high toxicity, and expensive price of these drugs make it still difficult for many MDR-TB patients. In some cases, more severe extensively drug-resistant TB may occur, and it will not respond to the most effective second-line anti-TB drugs (Sotgiu et al., 2015; Jeon, 2017; Tiberi et al., 2018). Obviously, the development of new anti-TB drugs is urgent, and exploring the resistance mechanism of rifampicin is of great significance for the discovery of effective drugs.

In this work, in order to uncover the resistance mechanism of Mtb to rifampicin due to the mutation of Mtb-RNAP at position 451, three independent molecular dynamics (MD) simulations for the wild-type Mtb-RNAP and H451D/Y/R mutants were carried out. Based on the obtained trajectories, the molecular mechanics generalized-Born surface area (MM-GBSA) method was applied to calculate the binding free energy of rifampicin with Mtb-RNAP. Furthermore, dynamic network analysis combined with residue interaction network (RIN) analysis was used to show the detailed changes of interactions among the residues surrounding the binding pocket. With the structural and energy analysis, a possible rifampicin-resistant mechanism was also proposed. Compared with the traditional experimental method, MD simulations can show the intuitive and dynamics interaction change process between rifampicin and Mtb-RNAP due to the point mutation. Together with the energy analysis and the dynamics network analysis, the present study show the essential reason of Mtb-RNAP resistant to rifampicin, which can provide the useful guidance for the further drug design against drug resistance.

## Materials and Methods

### Systems Preparation

**Figure 1:**
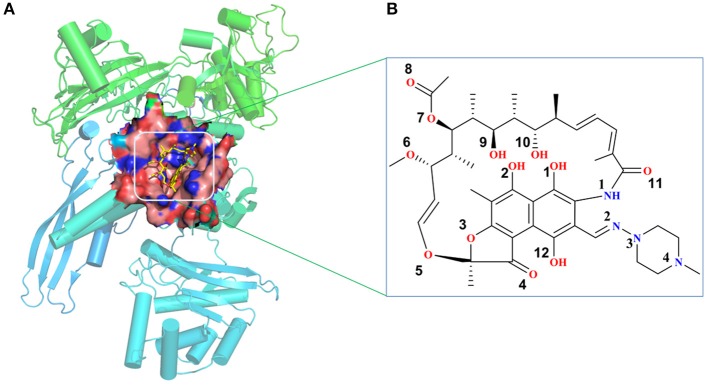
**(A)** The structural overview for *Mycobacterium tuberculosis* RNA polymerase bound with rifampicin (the protein is shown as cartoon, rifampicin is shown as a stick, the binding pocket of rifampicin is shown as surface). **(B)** The molecular scheme of the rifampicin.

The initial atomic coordinate of the wild-type Mtb-RNAP with rifampicin was obtained from Protein Data Bank (Protein Data Bank ID: 5UHB). The crystal structure of Mtb-RNAP reported by Lin et al. (2017) reveals that Mtb-RNAP is composed of six chains, for the A, B chains encoded by the rpoA gene, the C chain encoded by the rpoB gene (Miller et al., 1994), and the D, E, F chains encoded by rpoC, rpoZ, and rpoD, respectively. Rifampicin binds at the active site of the C chain (shown in Figure 1) and inhibits the DNA-directed RNA synthesis of Mtb (McClure and Cech, 1978; Campbell et al., 2001; Somoskovi et al., 2001). Considering that the speed to simulate the whole Mtb-RNAP (~3,826 residues) is too slow, only the C chain complexed with rifampicin was extracted and used as the initial structure of simulations. Furthermore, the deletion of other chains will make the residues of the interface between the two chains unstable, which is inconsistent with that in the multimer. Thus, to simulate the state of interface in the multimer, some relatively flexible and far from the active site amino acid residues were deleted. The three-dimensional structures of three mutants (H451D/Y/R) were obtained by mutating H451 residue in wild type.

To generate the force field parameters for the ligand, the Gaussian 09 program (Frisch et al., 2009) was used to optimize the structure of rifampicin and calculate the electrostatic potential at the Hartree–Fock level with 6-31G^*^ basis set. Then, the restraint electrostatic potential protocol (Bayly et al., 1993; Cieplak et al., 1995; Fox and Kollman, 1998) was employed to fit the atomic partial charges. The general amber force field (gaff) (Wang et al., 2004) generated by the antechamber program in the Amber14 package (Case et al., 2014) was applied to describe the ligand. The standard ff99SB force field (Hornak et al., 2006) was used to describe the protein. Then, the LEaP module was applied to add all missing hydrogen atoms and a certain amount of sodium counter-ions to neutralize the unbalanced charges and maintain the systems electro-neutrality. Finally, a rectangular periodic water box of TIP3P (Jorgensen et al., 1983) was added to each system with the water molecules extended 10-Å distance around the complex. The size of the periodic boundary box is 92.6 × 114.4 × 114.9 Å. The whole system has a total of ~100,000 atoms per periodic cell.

### Molecular Dynamics Simulations

All MD simulations were performed with Amber14 package (Case et al., 2014). The process of energy minimization, heating, and equilibration was carried out with the Particle Mesh Ewald Molecular Dynamics module. Initially, the energy minimization of each solvated complex includes three steps. For each step, energy minimization was carried out by the steepest descent method for the first 2,500 steps and conjugated gradient method for the subsequent 2,500 steps. In the first step, all the atoms of the complex were restrained with a force constant of 2.0 kcal/(mol·Å^2^) to only minimize the solvent and ion molecules. After that, the protein backbone atoms were fixed with a restraint force of 2.0 kcal/(mol·Å^2^) in the second step. Finally, all atoms in the system were minimized without any restraint. After energy minimization, all systems were heated up from 0 to 310 K in the canonical (NVT) ensemble over 100 ps by restraining the protein and the ligand with a 2.0 kcal/(mol·Å^2^) force constant and using a Langevin thermostat with a coupling coefficient of 2.0/ps. After heating, five steps MD pre-equilibration at 310 K were performed in the NPT ensemble by restraining all the atoms of the complex with decreasing restraints from 2.0 to 1.5, to 1.0, to 0.5, to 0.1. Then, 50-ns equilibration MD simulation without any restraints was performed to eliminate collisions between atoms. Finally, 500-ns production MD simulation was carried out without any restraints on each system in the NPT ensemble at the temperature of 310.0 K and pressure of 1 atm. During the simulations, all the bonds involving hydrogen atoms were restrained with SHAKE algorithm (Ryckaert et al., 1977) to avoid too fast vibration of the hydrogen atoms. In addition, periodic boundary conditions were employed, and the long-range Coulombic interactions were treated using the Particle Mesh Ewald (Darden et al., 1993). The time step was set to 2 fs.

### Molecular Mechanics Generalized-Born Surface Area Calculation

To uncover the effects of mutations on the binding affinity of rifampicin to Mtb-RNAP, the MM-GBSA method was applied to estimate the binding free energy of each complex, which has been successfully used in a lot of researches (Pan et al., 2011; Yang et al., 2012). Here, we extracted 1,000 snapshots at 100-ps interval from the last 100-ns trajectory for each system. The binding free energy was calculated from the equation:

(1) $$\begin{array}{l} \Delta {\text{G}}_{bind}={\text{G}}_{complex}-{\text{G}}_{receptor}-{\text{G}}_{ligand} \end{array}$$

where G_*complex*_, G_*receptor*_, and G_*ligand*_ are the free energy of complex, protein, and ligand, respectively. The free energy for each molecular species was calculated based on an average over the extracted snapshots. Each of them can be estimate with the following equations:

(2) $$\begin{array}{l} \text{G}={\text{E}}_{MM}+{\text{G}}_{sol}-\text{TS} \end{array}$$

(3) $$\begin{array}{l} {\text{E}}_{MM}={\text{E}}_{int}+{\text{E}}_{ele}+{\text{E}}_{vdw} \end{array}$$

(4) $$\begin{array}{l} {\text{E}}_{int}={\text{E}}_{bond}+{\text{E}}_{angle}+{\text{E}}_{torsion} \end{array}$$

(5) $$\begin{array}{l} {\text{G}}_{sol}={\text{G}}_{GB}+{\text{G}}_{SA} \end{array}$$

(6) $$\begin{array}{l} {\text{G}}_{SA}=\gamma^{*}\text{SASA}+\beta \end{array}$$

where E_*MM*_ is the gas-phase energy calculated using the Amber ff03 molecular mechanics force field. E_*int*_ is the internal energy, including the energy of bond (E_*bond*_), angle (E_*angle*_), and torsion (E_*torsion*_). E_*ele*_ and E_*vdw*_ are the Coulomb and van der Waals energy, respectively. G_*sol*_ is the solvation free energy and can be decomposed into polar solvation free energy (G_*GB*_) and non-polar solvation free energy (G_*SA*_). G_*GB*_ was calculated by solving the GB equation and the dielectric constants for solute as well as solvent were set to 1.0 and 80.0, respectively (Rocchia et al., 2001). G_*SA*_ was estimated by the solvent accessible surface area determined using a water probe radius of 1.4 Å. The surface tension constant γ was set to 0.0072 kcal/(mol·Å^2^), and the non-polar contribution to the solvation free energy term β was set to 0 (Sitkoff et al., 1994). T and S are the temperature and the total solute entropy, respectively. The entropy contributions can be estimated by normal mode analysis (Pearlman et al., 1995). However, here, we did not calculate the entropy contributions since our aim is not to obtain the absolute Gibbs energy but to identify the key residues of binding pocket and the detailed interaction features. In addition, previous studies have proven that it is sufficient to compare the binding ability of receptors and ligands based on the values of enthalpy changes (ΔH_*bind*_) (Aruksakunwong et al., 2006; Xue et al., 2012).

Moreover, in order to identify the key residues responsible for the binding of rifampicin, the MM-GBSA binding free energy decomposition process was used to decompose the interaction energy to each residue by considering molecular mechanics and solvation energy without considering the contribution of entropy.

### Dynamic Network Analysis

Dynamic network analysis, as an effective method to extract information from the obtained molecular dynamics trajectories, has been successfully applied in protein misfolding (Zhou et al., 2019) and protein–protein interaction analysis (Sethi et al., 2009; Bai et al., 2014). Here, in order to observe the dynamic changes of the residues interaction network, 2,000 snapshots were extracted from the last 50-ns trajectory for each system. In the network, one node represents one residue, and the position of each node is defined at the center of Cα atom of residue. The edge represents the interactions of two residues. Furthermore, the edge weight (*W*_*ij*_) between two nodes (*i, j*) was defined with the following equation:

(7) $$\begin{array}{l} {\text{W}}_{ij}=-\log\;\left(\;|{\text{C}}_{ij}|\;\right) \end{array}$$

where *C*_*ij*_ represents the pairwise correlations, which is calculated by Carma program (Glykos, 2006), a plugin in VMD (Humphrey et al., 1996). Finally, the NetworkView module in VMD was used to visualize the residue interaction network.

### Residue Interaction Network Analysis

RIN uses a network diagram to simplify the inter-residue interaction, which considers the residues as nodes and physico–chemical interactions such as covalent and non-covalent bonds as edges. RIN method has been successfully used to analyze the effects of mutations on drug resistance (Xue et al., 2012, 2013, 2014). In this work, the residue interaction network generator 2.0 (RING-2.0) (Piovesan et al., 2016) software was applied to generate the network for the representative structures. The calculation process of RING-2.0 is described as follows: (i) the calculation of the secondary structure elements by incorporating the DSSP algorithm (Kabsch and Sander, 1983); (ii) hydrogen atom placement based on geometric criteria; (iii) hydrogen bond calculation; and (iv) the calculation of van der Waals interactions. Moreover, Cytoscape (Shannon et al., 2003) and the plugin RINalyzer (Doncheva et al., 2011) were used to visualize the residue interaction network.

## Results

### H451D/Y/R Mutations Increased the Flexibility of the Active Pocket

**Figure 2:**
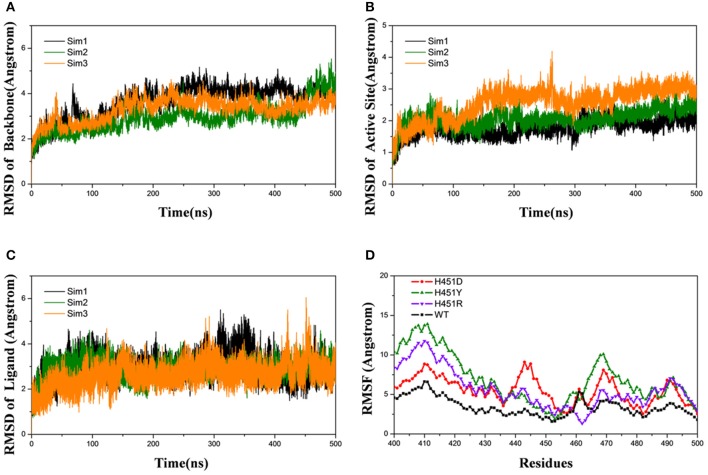
Root-mean-square-deviations (RMSDs) for the wild-type system of three independent molecular dynamics simulations: Sim1 (black), Sim2 (green), Sim3 (orange): **(A)** RMSDs for the backbone atoms of protein versus time. **(B)** RMSDs for the backbone atoms of active pocket vs. time. **(C)** RMSDs for the heavy atoms of rifampicin vs. time. **(D)** Root-mean-square-fluctuation for the backbone atoms of active pocket vs. residue number of wild type (black), H451D (red), H451Y (green), and H451R (purple) in Sim1.

Firstly, the root-mean-square-deviations (RMSD) value for the protein backbone atoms, the active pocket, and the heavy atoms of rifampicin relative to the initial structure were calculated to monitor the equilibrium of each system. As shown in Figure 2 and Supplementary Figures 1–3, three parallel MD simulations have similar fluctuations, suggesting each parallel trajectory can produce reproducible results. Thus, the following analysis was based on one of three parallel MD simulations. As can be seen from the RMSDs of wild-type Mtb-RNAP and three mutants, each system achieves equilibrium after 100 ns. Therefore, the last 100-ns trajectory for each system was used for the following structural and energetic analysis. Additionally, from the monitoring of the RMSD value of the heavy atoms of ligand, we can justify roughly if the ligand can bind to the target stably. From Figure 2C and Supplementary Figures 1–3 the RMSDs of rifampicin in mutants were larger than that in wild type, indicating that rifampicin had a large fluctuation in mutants.

The root-mean-square-fluctuation (RMSF) of each residue was calculated based on the last 100-ns trajectory for each system, and the corresponding results were shown in Figure 2D. It can be seen from Figure 2D that the RMSF values of the residues have similar trends for all systems. However, H451D/Y/R mutations will increase the RMSF values relative to the wild-type Mtb*-*RNAP, which indicates that these three mutations increased the flexibility of the binding pocket and weakened the interaction of rifampicin with Mtb-RNAP.

### H451D/Y/R Mutations Weaken the Binding Ability of *Mycobacterium tuberculosis* RNA Polymerase With Rifampicin

To explore the effects of three mutations on the binding of Mtb-RNAP with rifampicin, the binding free energy calculation was performed based on MM-GBSA method. As shown in Table 1, the enthalpy changes (ΔH_*bind*_) of the wild-type Mtb*-*RNAP and H451D/Y/R mutants with rifampicin are −43.89, −31.20, −35.55, and −28.58 kcal/mol, respectively. As expected, the binding affinity of Mtb*-*RNAP to rifampicin reduced obviously due to H451D/Y/R mutations.

**Table 1:** The calculated binding free energy and the detailed contribution of different energy terms (kcal/mol).

**Contribution**	**Wild type**	**H451D**	**H451Y**	**H451R**
ΔE*_*ele*_*	−64.92 ± 0.17	−26.97 ± 0.21	−47.82 ± 0.27	−23.79 ± 0.22
ΔE*_*vdw*_*	−52.49 ± 0.12	−45.07 ± 0.16	−50.01 ± 0.16	−46.03 ± 0.15
ΔE*_*MM*_*^a^	−117.41 ± 0.19	−72.04 ± 0.26	−97.84 ± 0.35	−69.83 ± 0.27
ΔG*_*SA*_*	−6.62 ± 0.01	−5.34 ± 0.02	−6.23 ± 0.01	−5.70 ± 0.02
ΔG*_*GB*_*	80.14 ± 0.14	46.18 ± 0.17	68.52 ± 0.23	46.94 ± 0.19
ΔG*_*sol*_*	73.52 ± 0.14	40.84 ± 0.16	62.29 ± 0.22	41.24 ± 0.18
ΔG*_*polar*_*^b^	15.22 ± 0.11	19.21 ± 014	20.7 ± 0.18	23.15 ± 0.14
ΔG*_*nonpolar*_*^c^	−59.11 ± 0.06	−50.41 ± 0.08	−56.24 ± 0.08	−51.73 ± 0.08
ΔH*_*bind*_*	−43.89 ± 0.12	−31.2 ± 0.17	−35.55 ± 0.22	−28.58 ± 0.16
MIC (mg/L)^d^	0.25–0.5	>8.0	>8.0	>8.0

a ΔE*_MM_ =* Δ*E_ele_ + ΔE_vdw_*.

b Δ*G_polar_ =* Δ*E_ele_ + ΔG_GB_*.

c Δ*G_nonpolar_ =* Δ*E_vdw_ +ΔG_SA_*.

d *In vitro activity of rifampicin in wild-type and rifampicin-resistant Mycobacterium tuberculosis*.

By assessing the contributions of individual energy terms, we found that the non-polar interactions (sum of van der Waals interaction energy ΔE_*vdw*_ and non-polar interaction energy in solvation free energy ΔG_*SA*_) are the driving force for the binding of rifampicin. However, the energy contributions of E_*vdw*_ and ΔG_*SA*_ decrease in the mutants. Relative to non-polar interactions, the polar interaction (sum of electrostatic interaction energy ΔE_*ele*_ and polar interaction energy in solvation free energy ΔG_*GB*_) seems like unfavorable for the binding of rifampicin and more apparent in the mutants (15.22, 19.21, 20.70, and 23.15 kcal/mol for the wild type, H451D, H451Y, and H451R mutants, respectively). Although the contributions of intermolecular electrostatic interactions (ΔE_*ele*_) are very favorable, their contributions are compensated by the large desolvation penalties.

#### Key Residues Responsible for the Reduced Binding Ability of H451D/Y/R Mutants

**Figure 3:**
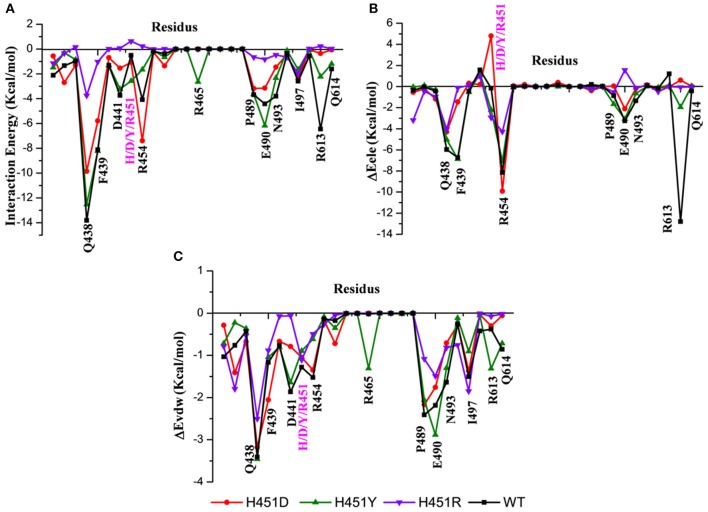
**(A)** The total energy contributions of the key residues for binding of rifampicin: wild type (black), H451D (red), H451Y (green), H451R (purple). **(B)** The electrostatic energy contributions of the key residues for binding of rifampicin. **(C)** The van der Waals energy contributions of the key residues for binding of rifampicin.

By decomposing the binding free energy of the wild-type Mtb-RNAP with rifampicin, 10 key residues such as Q438, F439, D441, R454, P489, E490, N493, I497, R613, and Q614 (Figure 3A) with energy contributions over 2 kcal/mol are identified. It can be seen that relative to the wild-type Mtb-RNAP, the contributions of Q438, F439, D441, E490, N493, R613, and Q614 have an obvious decrease in H451D mutant. For H451Y mutant, the reduced energy contribution of residues Q438, D441, R454, N493, R613, and Q614 should be responsible for the reduced binding affinity of rifampicin to Mtb-RNAP. Moreover, the energy reduction in H451R mutant is more obvious relative to that in H451D/Y mutants. Actually, the profile of each residue's energy contribution in three mutants shares some similar features. For example, in all three mutants, the residues Q438, D441, N493, R613, and Q614 have obvious reduced contribution for the binding of rifampicin, suggesting that the drug-resistance mechanisms due to three mutations have some similarities.

To further explore the origin of the reduced residues' contribution, we compared the electrostatic contribution and van der Waals contribution of the key residues (shown in Figures 3B,C, respectively). From Figure 3B, we can obtain when H451 is mutated to D451, the electrostatic energy contribution of D451 (4.80 kca/mol) is detrimental for rifampicin binding. Moreover, the contributions of electrostatic energy of Q438, F439, E490, and R613 decrease after H451D mutation. For H451Y mutant, the electrostatic energy contribution of three residues (Q438, R454, and R613) also have an obvious reduction. In H451R mutant, the contributions of electrostatic interactions of almost all key residues reduced. In addition, Figure 3C shows that the reduction of energy contribution of D441, P489, E490, N493, and Q614 is mainly from the loss of the van der Waals interaction contribution in H451D/Y/R mutants.

#### The Dynamic Network Analysis and Residue Interaction Network Analysis Reveal That H451D/Y/R Mutations Weaken the Interaction of Mutated Residue With Its Adjacent Residues

**Figure 4:**
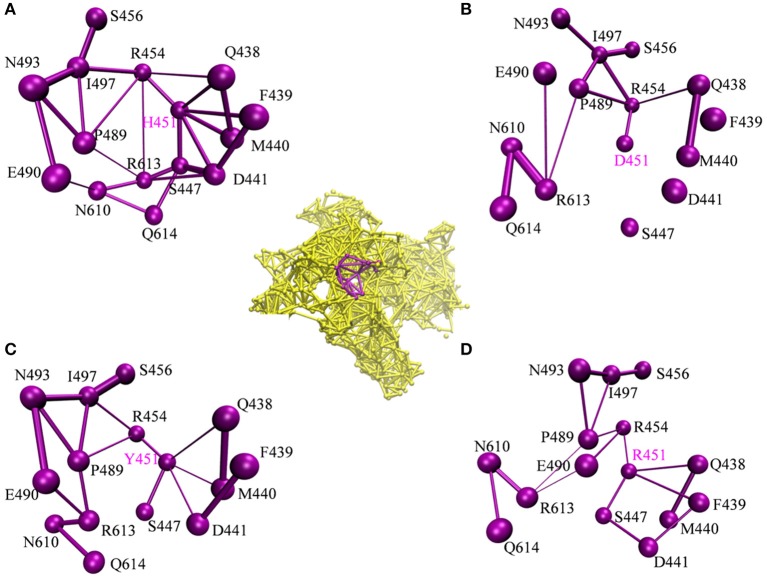
The picture of dynamic network analysis for the active pocket of rifampicin binding: **(A)** WT; **(B)** H451D; **(C)** H451Y; **(D)** H451R; The purple spheres represent the residues and the sticks represent the total interactions. The strength of interactions of two residues is indicted by the thickness degree of stick, the thicker of the stick, and the stronger interaction of two residues.

**Figure 5:**
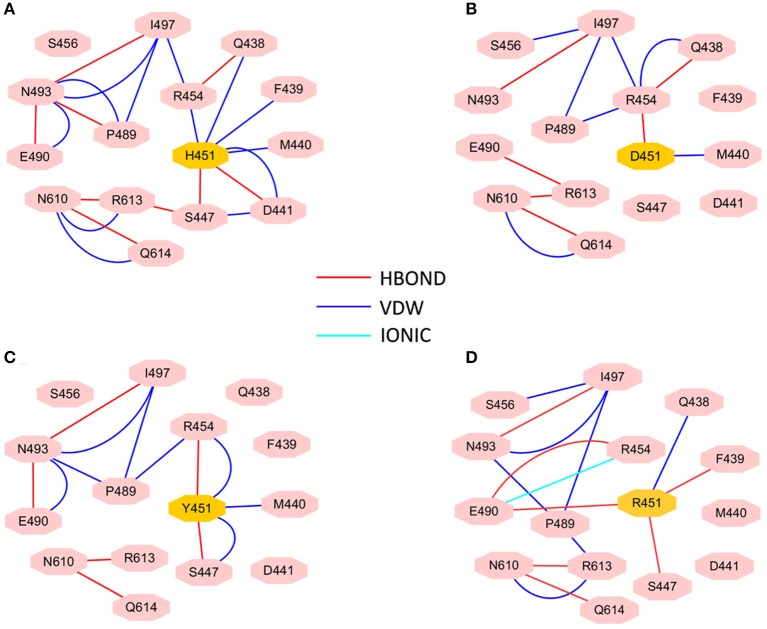
RIN for the active pocket of rifampicin binding **(A)** wild type; **(B)** H451D; **(C)** H451Y; **(D)** H451R. The pink octagons represent residues, and the yellow octagons represent the mutated residues; the edges represent van der Waals (blue) and hydrogen bond (red) interactions and salt bridge interaction (cyan).

To investigate how H451D/Y/R mutations change the binding pocket and further lead to the resistance of Mtb*-*RNAP to rifampicin, the dynamic network analysis was further carried out based on the 2,000 snapshots extracted from the equilibrium phase for each system. The obtained results are shown in Figure 4. The strength of the total interactions (including van der Waals interaction and hydrogen bond interaction and so on) is indicated by the edge thickness. Moreover, the type of interactions is shown by the two-dimensional RIN interactively based on the representative structure of each system (Figure 5). Here, the representative structures were extracted by clustering analysis, and the conformation with the lowest RMSD to the cluster center was selected.

In the wild-type Mtb*-*RNAP, H451 residue can be identified as the central node with six first neighbors in the interaction network according to Figures 4A, 5A. H451 not only can form strong van der Waals interactions with R454, Q438, F439, M440, and D441 but also form hydrogen bonds with D441, S447. Moreover, S447 acts as a key node in the network; in addition to forming hydrogen bond with H451, it also can form strong interactions with D441 (van der Waals interaction), R613 (hydrogen bond interaction), and Q614. I497, as another key node in the other side of the network, can form van der Waals interactions with R454, P489, and N493. Finally, R454 as a joint node can connect the two sides of the networks together by forming the van der Waals interactions with I497, H451, and the hydrogen bond with Q438. In addition, the weak “triangular shape” interaction network formed by R454, R613, and P489 residues that make the binding pocket more compact and coherent.

However, in H451D mutant, it is evident that the mutation causes the disappearance of the interactions between D451 and Q438, F439, M440, D441, and S447 mentioned previously (Figures 4B, 5B). For H451Y/R mutants, the corresponding interactions are also obviously weakened (Figures 4C,D). Overall, in H451D/Y/R mutants, the “triangular shape” interaction network is broken. Based on these results, it can be concluded that the mutations on 451 indeed reduced the interaction connection of the residues in the binding pocket and then result in the active pocket more flexible and open.

### The Comparison of Binding Modes of Rifampicin With the Wild-Type *Mycobacterium tuberculosis* RNA Polymerase and Three Mutants

**Figure 6:**
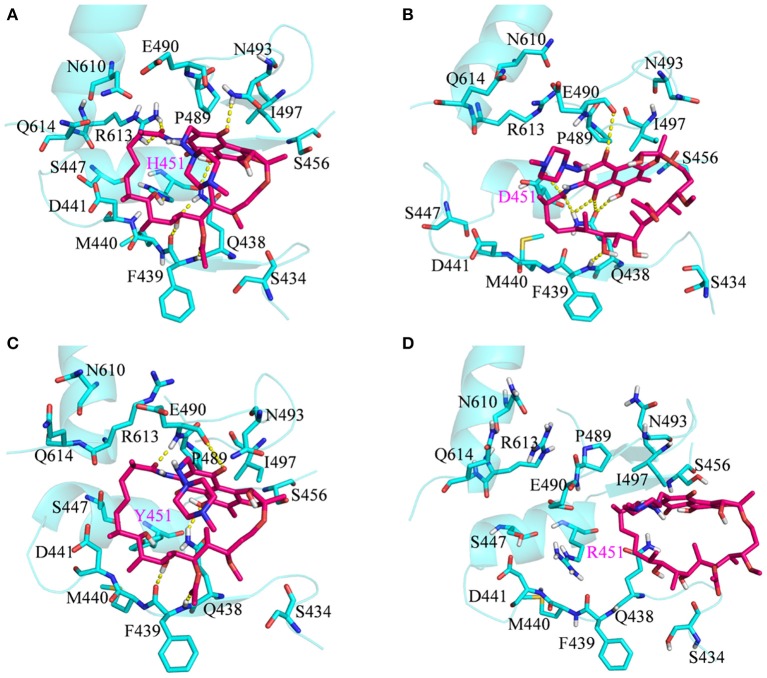
Three-dimensional representation for the binding mode of rifampicin with wild-type *Mycobacterium tuberculosis* RNA polymerase and three mutants based on the obtained representative structure: **(A)** wild type; **(B)** H451D; **(C)** H451Y; **(D)** H451R. The protein is shown as a cyan cartoon; rifampicin and the key residues are represented as hotpink and cyan carbon sticks, respectively; the intermolecular hydrogen bonds are indicated as dashed yellow lines.

To show the detailed rifampicin-resistance mechanism to Mtb-RNAP H451D/Y/R mutants intuitively, further structural analysis was performed. The representative structures are depicted in Figure 6. As shown in Figures 3C, 6A, the van der Waals interactions of Q438, D441, H451, R454, P489, E490, N493, I497, and Q614 with rifampicin are pivotal for the binding of rifampicin to the wild-type Mtb-RNAP. In addition, the calculation of hydrogen bond occupancy was carried out to monitor the formation of hydrogen bonds between rifampicin and Mtb-RNAP over the whole MD simulations. Based on the result in Table 2, we can see that the O8, O11, O9/O2, and O12 atoms of rifampicin can form stable hydrogen bonds with the side chains of Q438, F439, and R613 with high occupancy rate. The formation of these hydrogen bonds makes F439, R613, and Q438 have large electrostatic energy contributions (Figure 3B).

**Table 2:** The occupancy (%) of hydrogen bonds between rifampicin and *Mycobacterium tuberculosis* RNA polymerase.

**Complex**	**Donor**	**Acceptor**	**Distance (Å)**	**Angle (^**°**^)**	**Occupancy (%)^a^**
WT	RIF (O9-H6)	Phe439 (O)	2.72	166.09	100.00
	Phe439 (N-H)	RIF (O8)	2.90	163.84	99.92
	Arg613 (NH2-HH22)	RIF (O11)	2.83	158.70	99.82
	Gln438 (NE2-HE22)	RIF (O9)	2.97	158.79	96.27
	Asn493 (ND2-HD21)	RIF (O12)	3.02	147.36	81.44
	Gln438 (NE2-HE21)	RIF (O2)	3.16	156.40	81.37
	Arg613 (NH1-HH12)	RIF (O11)	3.14	137.22	49.60
H451D	Phe439 (N-H)	RIF (O10)	2.30	160.76	98.07
	Arg454 (NH1-HH12)	RIF (O11)	2.89	149.16	86.45
	Gln438 (NE2-HE21)	RIF (O11)	2.30	150.29	77.69
H451Y	Phe439 (N-H)	RIF (O8)	2.88	164.64	99.90
	RIF (O9-H6)	Phe439 (O)	2.70	164.10	99.83
	Gln438 (NE2-HE22)	RIF (O9)	2.30	160.02	97.25
	Glu490 (N-H)	RIF (O11)	3.05	163.01	78.20
	Gln438 (NE2-HE21)	RIF (O2)	3.20	157.50	55.74
	Tyr451(O-H)	RIF (O10)	2.86	158.02	50.49
H451R	Gln438 (NE2-HE21)	RIF (O11)	3.02	154.50	54.26
	RIF (O9-H6)	Ser434 (O)	2.69	162.18	53.93

a *Only hydrogen bonds that existed more than 50% of the time were shown*.

By comparing the binding modes of wild-type and H451D mutant (Figures 6A,B), the position of rifampicin has a clear movement. Combined with the results of RIN analysis (Figures 5A,B), H451D mutation causes the interactions of residue 451 between with some residues of binding pocket disappear, further causing the conformations of some amino acids (such as D441, P489, E490, N493, and Q614) that changed a lot. Finally, the residues D441, P489, E490, N493, and Q614 are away from rifampicin, causing the weakened van der Waals interaction of these residues. From Table 2, some hydrogen bonds between rifampicin and Q438, F439, and R613 disappeared in H451D mutant, which causes the reduction of the electrostatic energy contribution (Figure 3B). Moreover, the H451D mutation leads to the electrostatic repulsion between carboxyl group of D451 and O10 atom of rifampicin and is unfavorable for the binding of rifampicin.

For H451Y mutant, the binding mode of rifampicin with Mtb-RNAP is more similar to that in the wild-type Mtb-RNAP, and the position of rifampicin just has a slight movement (Figures 6A,C). Despite this, the mutation still causes some residues (such as N493, I497, and Q614) away from rifampicin, reducing the nonpolar contribution of these residues. In addition, the result in Table 2 shows that the disappearance of the hydrogen bond between R613 and rifampicin is responsible for the reduction of electrostatic contributions of R613. The reduction of hydrogen bond occupancy rate formed between Q438 and rifampicin causes the loss of electrostatic contribution of Q438.

**Figure 7:**
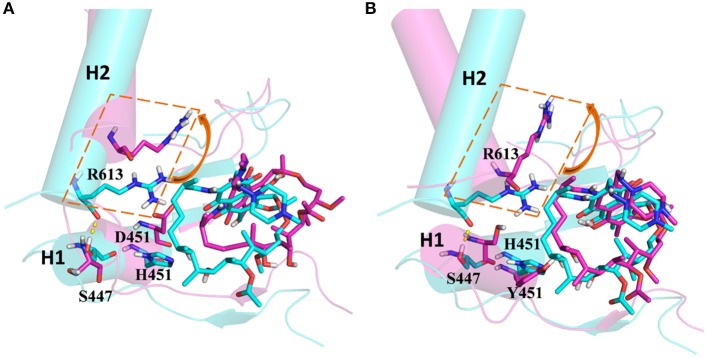
**(A)** The superimposition of wild-type *Mycobacterium tuberculosis* RNA polymerase and H451D mutant. **(B)** The superimposition of wild-type *Mycobacterium tuberculosis* RNA polymerase and H451Y mutant; the protein is shown as cartoon with cylindrical helices colored with cyan (wild type) and magenta (mutant); rifampicin and R613, S447, H451, D451, and Y451 are shown as stick colored with cyan (wild type) and magenta (mutant); the intermolecular hydrogen bond between S447 and R613 is indicated as dashed yellow lines.

From the energetic and structural analysis discussed previously, it can be seen that the flipping of R613 greatly affects the binding of rifampicin in H451D/Y mutants. Therefore, it is worthy to explore how the mutation of H451 affects the flipping of R613. For this aim, we superimpose the binding pocket of the wild-type Mtb-RNAP with those of H451D/Y mutants. Figure 7 shows the superimposition results. From Figure 7A, it can be seen that the position of H451 is on H1. When H451 is mutated to D451, no hydrogen bond can be formed between D451 and S447 (Figure 5B), which causes the helix structure of S447 to transform into disordered loops. At the same time, the fluctuation of this loop further causes the disappearance of the hydrogen bond between R613 (located on H2) and S447 (located on H1). Therefore, the R613 residue flips with H2 due to the weaker interaction between H1 and H2. However, the mutation of H451Y enhances the van der Waals interaction of Y451 and S447 (Figure 5C), which increases the distance of S447 and R613 and further to interfere the formation of hydrogen bond. Moreover, the rotation of H2 makes the flipping of R613 more obvious. The flipping of R613 directly causes the disappearance of the hydrogen bonds with rifampicin, further leading that the electrostatic contribution of R163 reduced.

**Figure 8:**
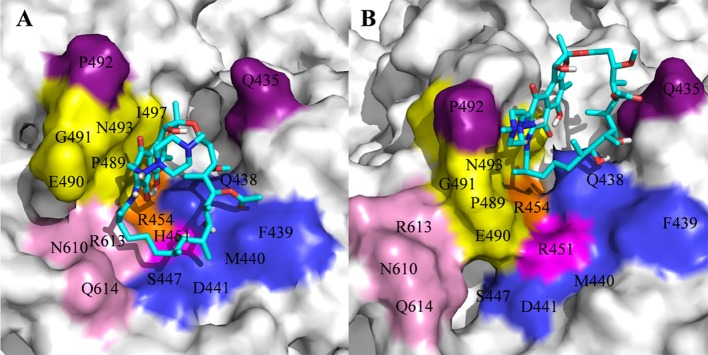
The binding pocket of rifampicin: **(A)** wild type; **(B)** H451R mutant; the pocket is shown as surface, and rifampicin is shown as stick colored with cyan.

For H451R mutant, there was a large conformational transition of the binding pocket and rifampicin as shown in Figure 6D. Moreover, R451 has a steric clash with rifampicin and so that rifampicin moves out of the binding pocket. Thereby, the movement of rifampicin causes the energy contribution of some key residues such as R613, E490, S447, D441, M440, and F439 reduce (Figure 3). In addition, some hydrogen bonds between rifampicin and F439/R613 also disappear (Table 2). In order to observe the changes of the binding pocket more intuitively, the surface map for the binding pocket of the wild-type and H451R mutant is depicted in Figure 8. It can be seen that when H451 is replaced by R451, the long side chain of R451 made the binding pocket smaller and cannot accommodate the relatively rigid rifampicin, which leads to the movement of rifampicin.

**Figure 9:**
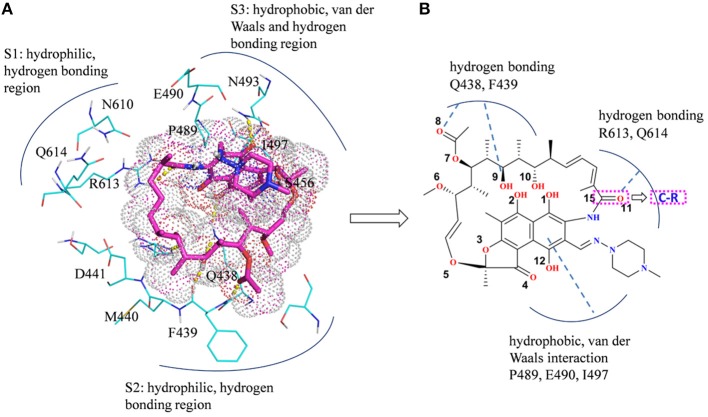
**(A)** Three main binding sites between rifampicin and the active pocket. Rifampicin is shown as stick colored with magenta, and the residues are shown as lines colored with cyan. The yellow dotted lines represent the hydrogen bond interactions. **(B)** Structural optimization of rifampicin. R represents a longer negatively charged group.

In summary, there are three main binding sites between rifampicin and the active pocket in wild type (Figure 9A). The polar pocket formed by the residues N610, R613, Q614, etc. (S1) can act as the hydrogen bond acceptor to form the hydrogen bond with the oxygen atom of rifampicin. The other polar pocket (S2) consists of residues Q438, M440, D441, etc., which also forms stable hydrogen bonds with rifampicin. In addition, the hydrophobic pocket composed of residues such as P489, N493, and I497 just accommodates the naphthalene ring of rifampicin. However, H451D/Y/R mutants changed the initial binding mode for rifampicin resulting from the change of the side chain size of 451. The N atom from imidazole ring of H451 can form the hydrogen bond interaction with rifampicin in wild-type RNA polymerase, which is interfered by these mutations. Furthermore, the mutations in 451 position impaired the interaction between the residue 451 and other key residues in the active pocket, interfering with the specific orientation of the key residues' side chains, thereby increasing the flexibility of the key residues such as R613. As a result, the binding affinity of rifampicin reduced, and the important interactions between rifampicin and active pocket were disturbed. One of the most apparent changes from the results of binding free energy decomposition was that the reduction of energy contribution from R613, which was mainly because the disappearance of the hydrogen bond between R613 and O atom at the C15 position of rifampicin (Figure 9B). Therefore, according to the obtained mechanism, to overcome the drug resistance induced by H451D/Y/R, one possible strategy is to enhance the interaction of the inhibitor with R613 by replacing the carbonyl group at the C15 position in rifampicin with a longer and negatively charged group (R). Such group may recover the hydrogen bond interaction between R613 and inhibitor, which could stabilize the binding of rifampicin.

## Conclusion

In this study, MD simulations together with MM-GBSA, dynamic network analysis, and RIN analysis were carried out on the complexes of rifampicin with the wild-type Mtb-RNAP and H451D/Y/R mutants to explore the resistant mechanism of rifampicin. The results from the MM-GBSA calculations are well-consistent with the experimental result. The reduced binding affinity for the studied mutants mainly comes from the loss of van der Waals contribution of D441, P489, E490, N493, I497, and Q614 and electrostatic contribution of Q438, F439, and R613 in mutants. The electrostatic energy contribution of R613 decreases obviously with the disappearance of the hydrogen bonds between R613 and rifampicin, which caused by the conformation flipping of R613 in H451D/Y mutants. The binding modes and dynamic network analysis show that the weakened interactions among D/Y/R451 with Q438, F439, M440, D441, and S447 increase the flexibility of the binding pocket, thereby reducing the binding affinity of rifampicin to Mtb-RNAP. In addition, these mutations caused the key hydrogen bond interactions between residue 451 and rifampicin disappear. Finally, the position of rifampicin had a clear movement, which changed the stable binding mode of rifampicin. We firstly proposed the atomic level resistance mechanism of Mtb to rifampicin due to H451D/Y/R mutations on Mtb-RNAP. In addition, we also proposed some guidance for the alteration of the rifampicin and the development of new drugs in the future. Though the hypothesis is still unvalidated on the traditional experimental, it can provide some theoretical underpinnings for the design of new anti-TB drugs to some extent, or a particular aspect.

## Data Availability Statement

All datasets generated for this study are included in the article/Supplementary Material.

## Author Contributions

QZ and HuL designed the research and wrote the manuscript. QZ, XA, HoL, SW, and TX performed the dynamic simulations and analyzed the data.

### Conflict of Interest

The authors declare that the research was conducted in the absence of any commercial or financial relationships that could be construed as a potential conflict of interest.
