# Supplementary material for: Uncovering the Resistance Mechanism of Mycobacterium tuberculosis to Rifampicin Due to RNA Polymerase H451D/Y/R Mutations From Computational Perspective
Source: Front Chem. 2019 Dec 3;7:819. doi: 10.3389/fchem.2019.00819 (PMC6902089; doi:10.3389/fchem.2019.00819)
Supplement: Supplementary file 1 [file Table_1.docx]

**Supplementary Material**

**Supplementary Figures**

**
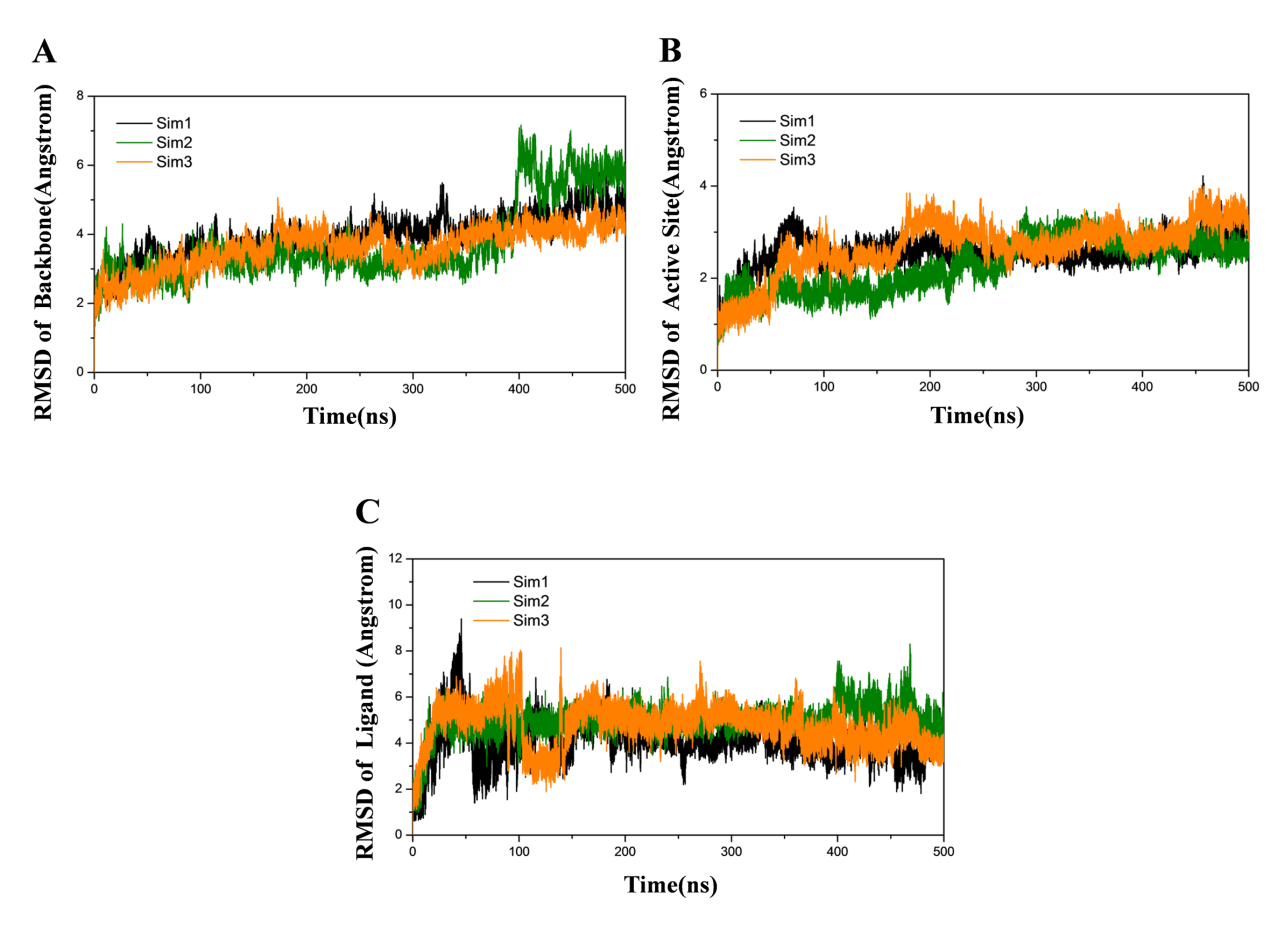
**

**Supplementary Figure 1.** Root-mean-square-deviations (RMSDs) for the H451D system of three independent MD simulations: Sim1 (black), Sim2 (green), Sim3 (orange): **(A)** RMSDs for the backbone atoms of protein versus time; **(B)** RMSDs for the backbone atoms of active pocket versus time; **(C)** RMSDs for the heavy atoms of rifampicin versus time.

**
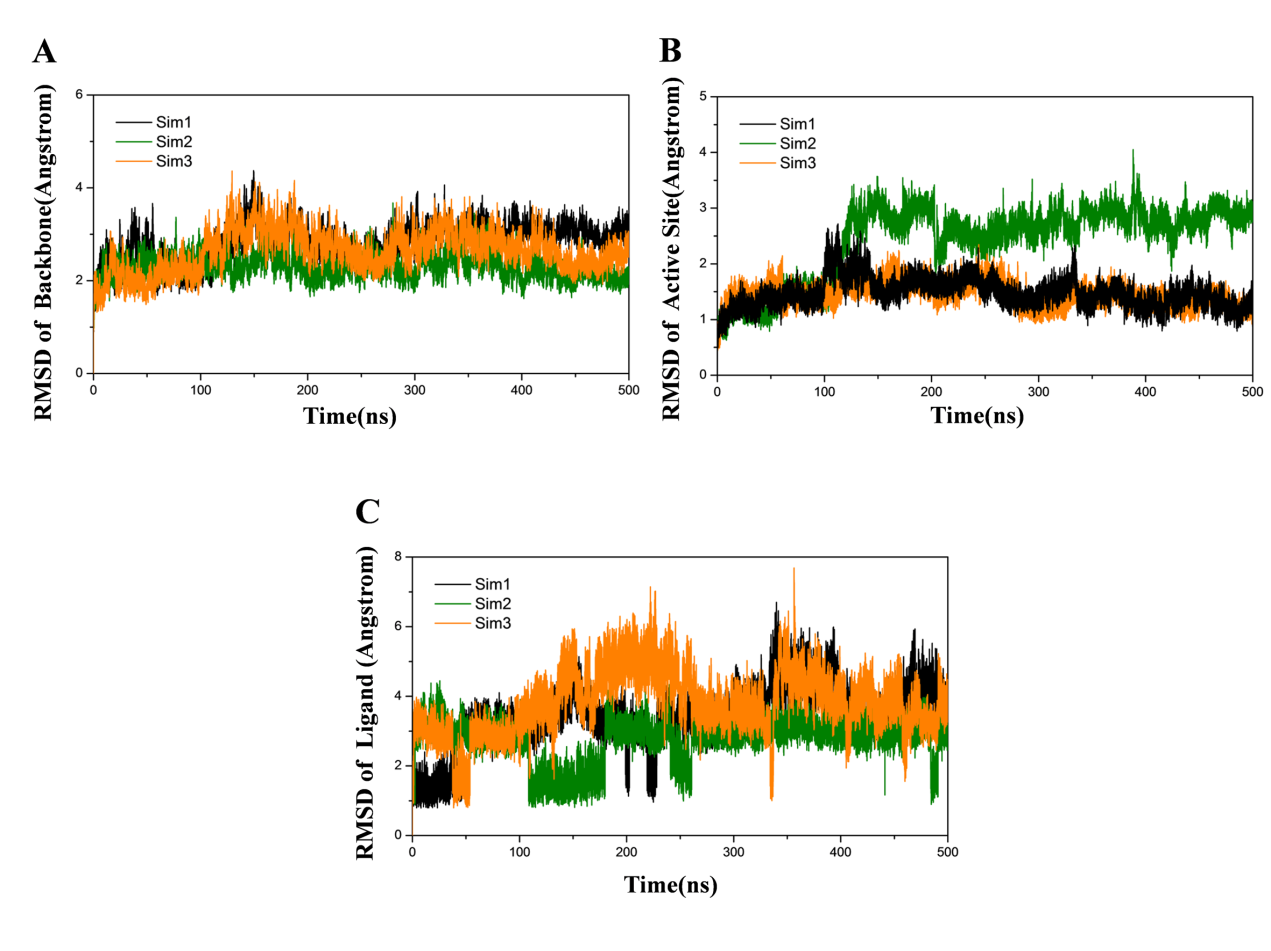
**

**Supplementary Figure 2.** Root-mean-square-deviations (RMSDs) for the H451Y system of three independent MD simulations: Sim1 (black), Sim2 (green), Sim3 (orange): **(A)** RMSDs for the backbone atoms of protein versus time; **(B)** RMSDs for the backbone atoms of active pocket versus time; **(C)** RMSDs for the heavy atoms of rifampicin versus time.

**
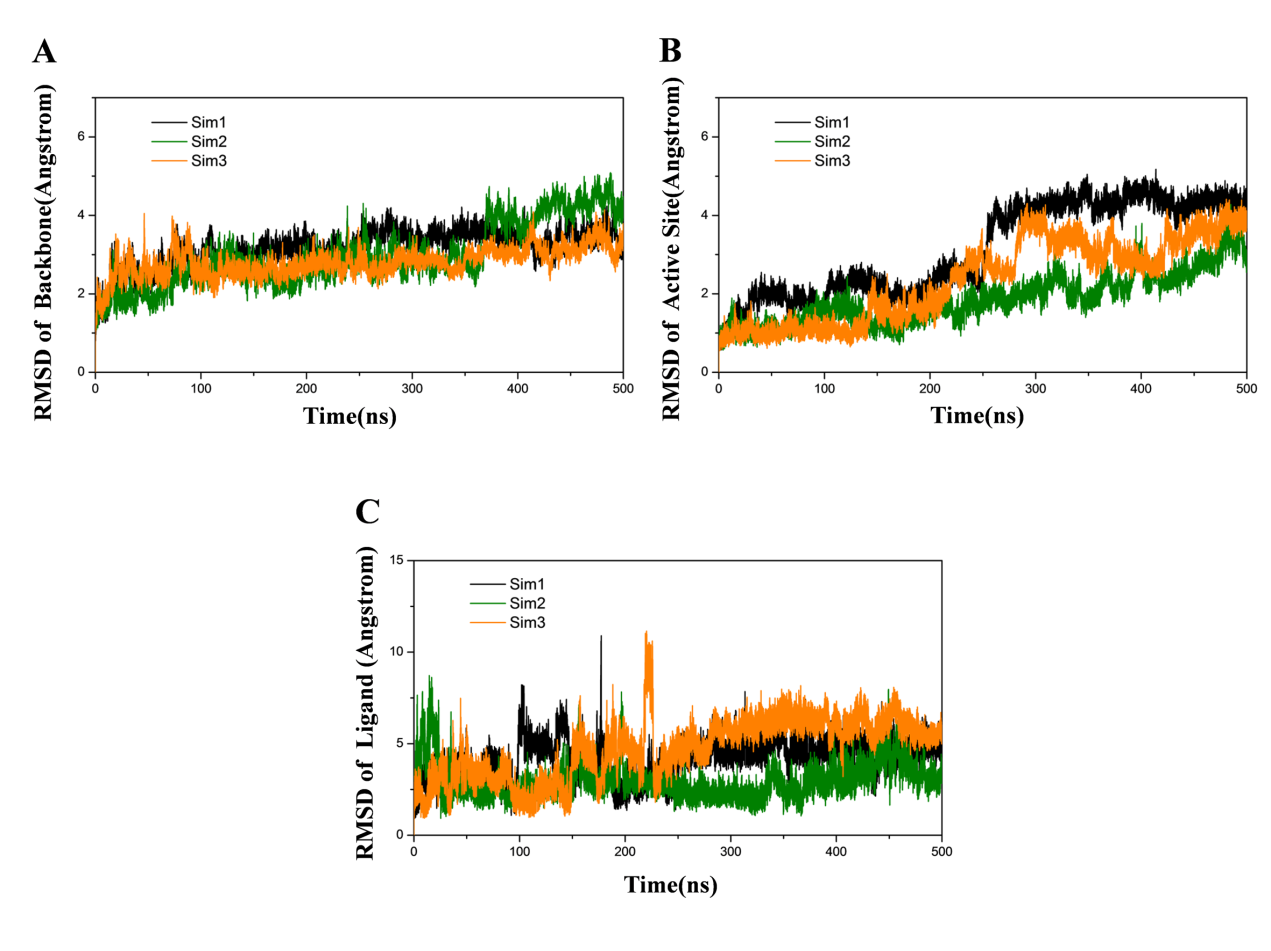
**

**Supplementary Figure 3.** Root-mean-square-deviations (RMSDs) for the H451R system of three independent MD simulations: Sim1 (black), Sim2 (green), Sim3 (orange): **(A)** RMSDs for the backbone atoms of protein versus time; **(B)** RMSDs for the backbone atoms of active pocket versus time; **(C)** RMSDs for the heavy atoms of rifampicin versus time.
